# Supplementary material for: Plant-nanoparticles enhance anti-PD-L1 efficacy by shaping human commensal microbiota metabolites
Source: Nat Commun. 2025 Feb 3;16:1295. doi: 10.1038/s41467-025-56498-2 (PMC11790884; doi:10.1038/s41467-025-56498-2)
Supplement: Supplementary file 1 — Supplementary Information [file 41467_2025_56498_MOESM1_ESM.pdf]

Supplementary Materials for

Plant-nanoparticles enhance anti-PD-L1 efficacy by shaping human commensal microbiota metabolites

Yun Teng[1, # *], Chao Luo[1,2, #], Xiaolan Qiu[1,3, #], Jingyao Mu[1], Mukesh K. Sriwastva[1], Qingbo Xu[4], Minmin Liu[1,3], Xin Hu[5], Fangyi Xu[1], Lifeng Zhang[1], Juw Won Park[1,6], Jae Yeon Hwang[1], Maiying Kong[1,6], Zhanxu Liu[1], Xiang Zhang[7], Raobo Xu[7], Jun Yan[1], Michael L Merchant[8], Craig J McClain[9] and Huang-Ge Zhang[1,4,10,11,*]

[#] These authors contributed equally
* Corresponding author: h0zhan17@louisville.edu (H.-G.Z.) and yun.teng@louisville.edu (Y.T.)

The PDF file includes:

Supplementary Figs. 1 to 10
Supplementary Tables 1 to 10

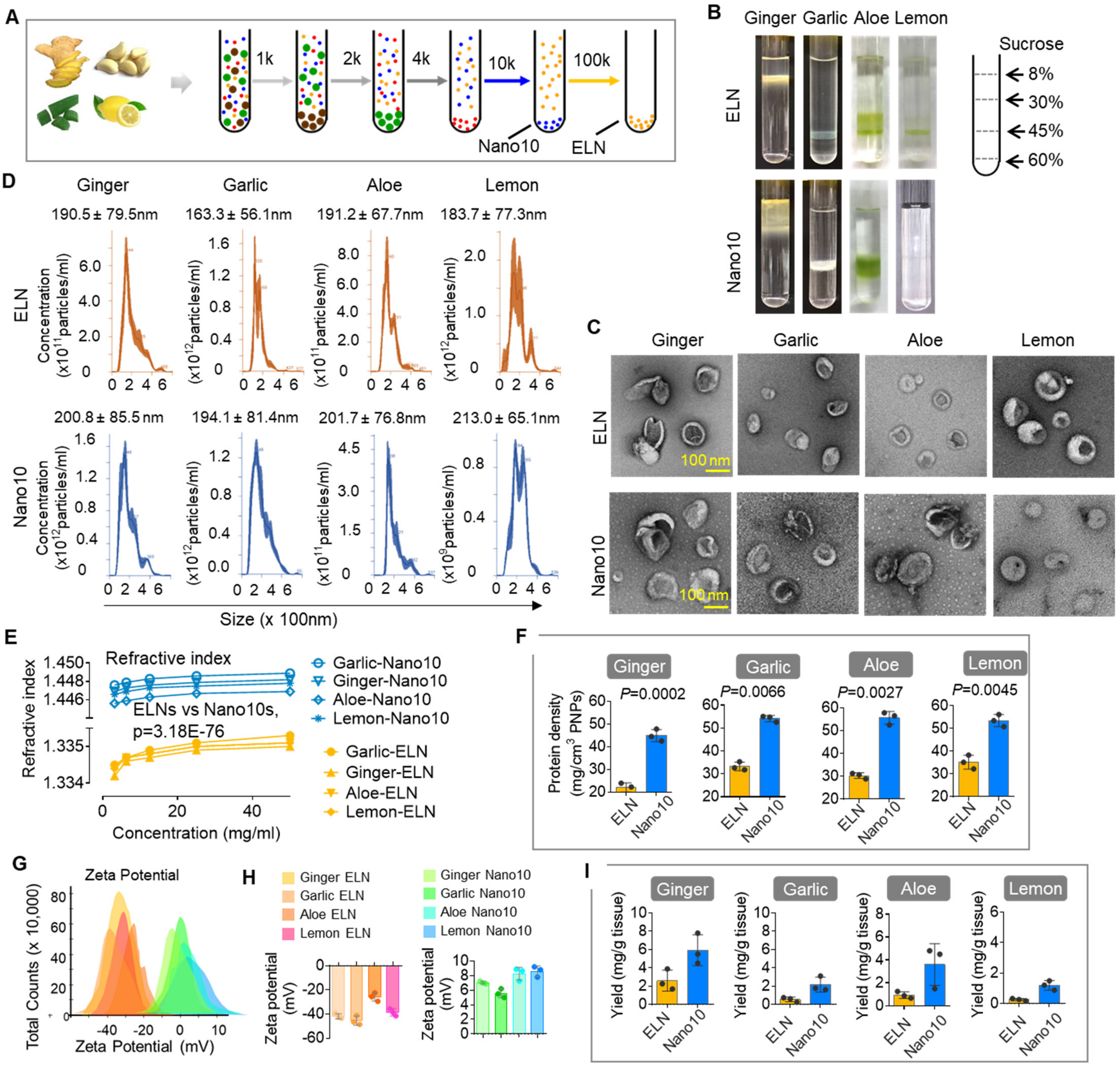

Supplementary Fig. 1 Isolation and characterization of plant-derived nanoparticles (PNPs). A Schematic diagram for PNP extraction from ginger, garlic, aloe and lemon by differential centrifugation. Nano10 and exosome-like nanoparticle (ELN) collected from the centrifugation at 10,000x g and 100,000x g, respectively, for 60 min at 4°C. **B** Purification of PNPs by sucrose gradient centrifugation with the concentration of sucrose indicated. **C** Representative electron microscopy image of PNPs. Scale bars, 100 nm. **D** Size distribution (Mean ± SD) of PNPs using a NanoSight NS300 (Westborough, MA) with a flow speed of 0.03 mL per min. **E** Refractive index of PNPs in different concentrations assessed using a refractometer. **F** PNPs protein extracted with CTAB lysis buffer and concentration determined with the BCA assays. (*P* = 0.0002, *P* = 0.0066, *P* = 0.0027, *P* = 0.0045, two-way t-test, n=3). **G** Representative graph of Zeta potential distribution of PNPs estimated using a ZetaView with the laser wavelength set at 520 nm. **H** Quantification of the Zeta potential of PNPs. **I** Quantification of PNP yield by weight. Data are representative of three independent experiments as the mean ± standard deviation (SD, error bars). Source data are provided as a Source Data file.

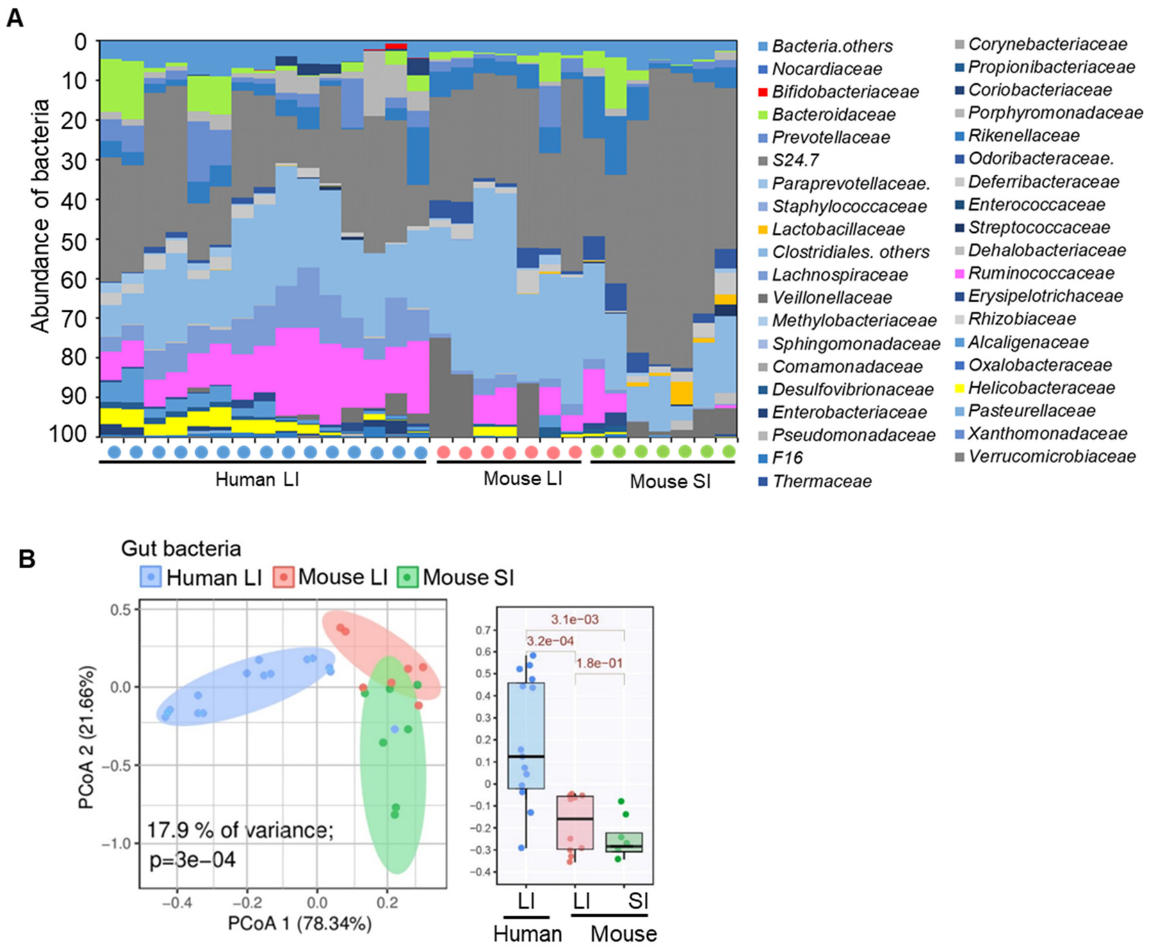

Supplementary Fig. 2 16s rRNA sequence analysis of gut bacteria in human intestine and germ-free (GF) mice colonized with human fecal bacteria (hFB). A Gut bacteria from human feces, hFB mice LI and SI. The species profiled with 16S rRNA gene sequencing compared with the taxonomy of OTUs generated from 16S sequencing at the family level. **B** PCoA of conventional gut bacteria. Source data are provided as a Source Data file.

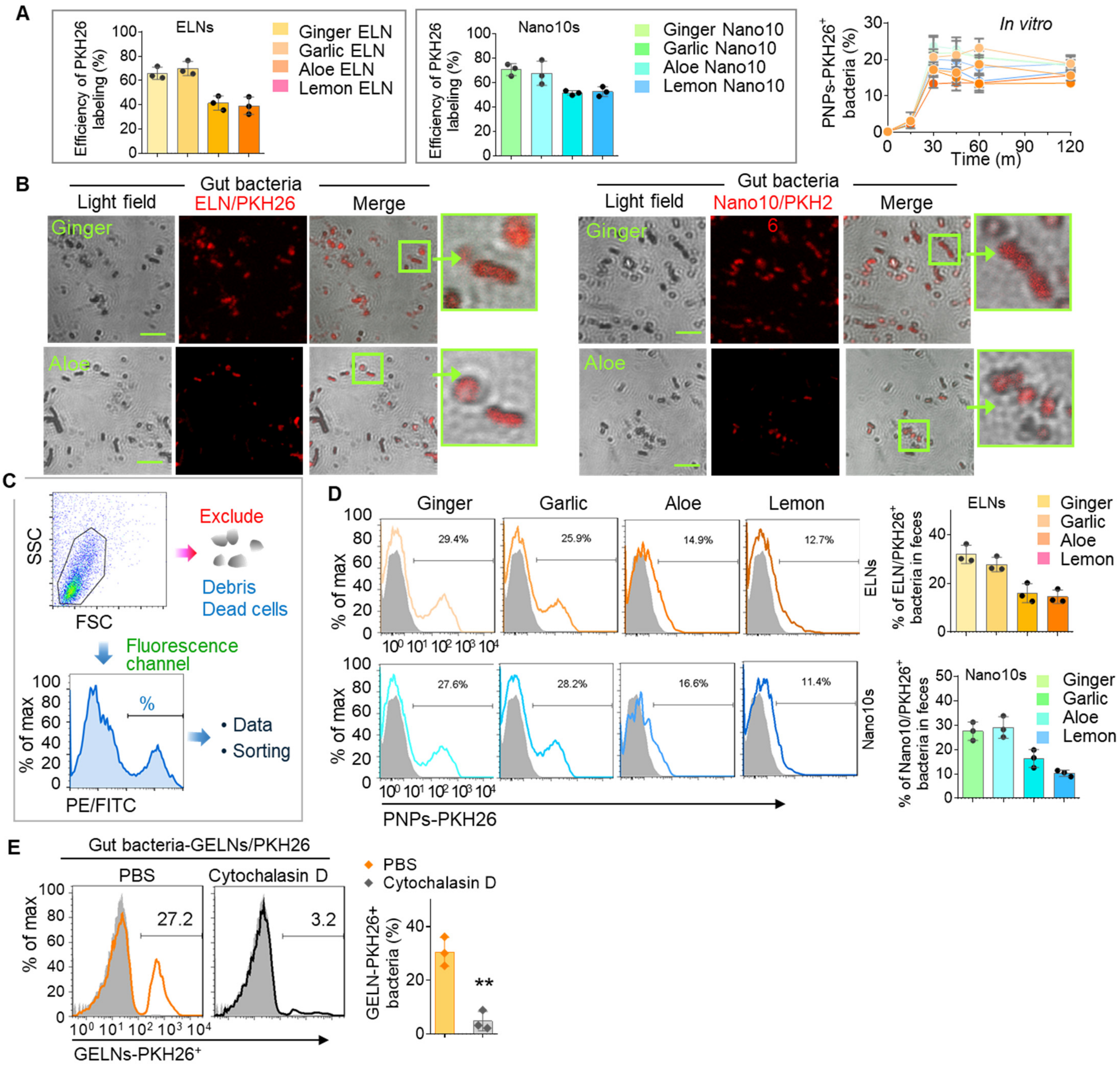

Supplementary Fig. 3 PNP uptake by human gut bacteria. A Bar graph shows the efficiency of PKH26 labeling on PNPs based on Nanosight analysis. Efficiency of labeling (%) = Nanoparticles-PKH26+/Total Nanoparticles. FACS analysis of gut bacteria uptake of PKH26-labeled PNP at different time points (right panel). **B** Representative confocal microscopy image of a fecal sample from human gut fecal bacteria (hFB) colonized mice fed PKH26-labeled ELNs (scale bar: 20 µm); **C** For FACS analysis, forward versus side scatter (FSC vs SSC) gating used to identify cells of interest while exclude debris and dead cells, followed by gating signals in fluorescence channels such as PE and FITC for further analysis of cell markers or collection of cells. **D** FACS analysis of ELNs/PKH26+ bacteria. Numbers above the bracketed lines and in quadrants indicate percent positive cells; gray, isotype-matched control antibody (left panel). Quantitative FACS analysis (right panel). **E** Bacteria treated with phagocytosis inhibitor cytochalasin D (10 µg/ml) for 1 h prior to be incubated with ginger ELNs (GELNs)-PKH26 in vitro following FACS analysis (left panel). Quantitative FACS analysis (right panel). (*P* = 0.0046, two-way Chi-Square test, n=3). Size based on diameter and concentration of GELN and Nano10 estimated using a NanoSight NS300. Data are representative of three independent experiments (mean ± SD). **p < 0.01 (two-tailed Chi-Square test). Source data are provided as a Source Data file.

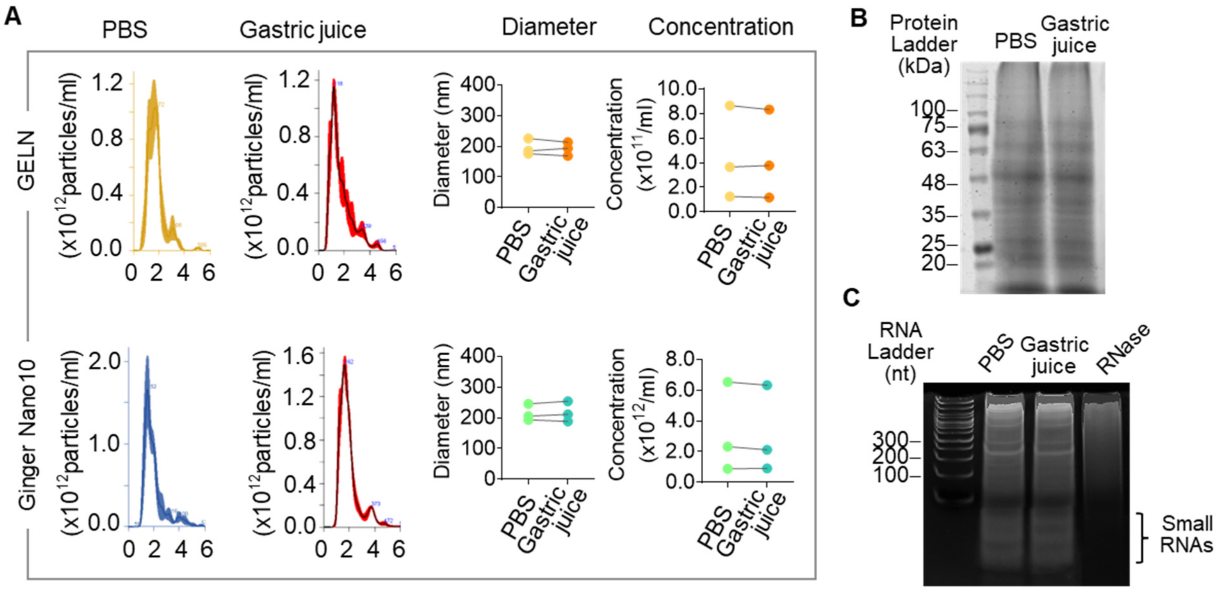

Supplementary Fig. 4 The stability analysis of PNPs in gastric juice. A GELN and ginger Nano10 were exposed to gastric juice ($1x10^{13}$/ml) collected from mice for 3 h at 37°C. Size based on diameter and concentration of GELN and Nano10 estimated using a NanoSight NS300. **B** Impact of gastric juice on the GELN protein using 10% SDS-PAGE gel staining with Coomassie brilliant blue **C** Impact of gastric juice on the GELN RNA using 10% denaturing PAGE gel staining by ethidium bromide. GELN RNA removed by RNase used as control. Data are representative of three independent experiments. Source data are provided as a Source Data file.

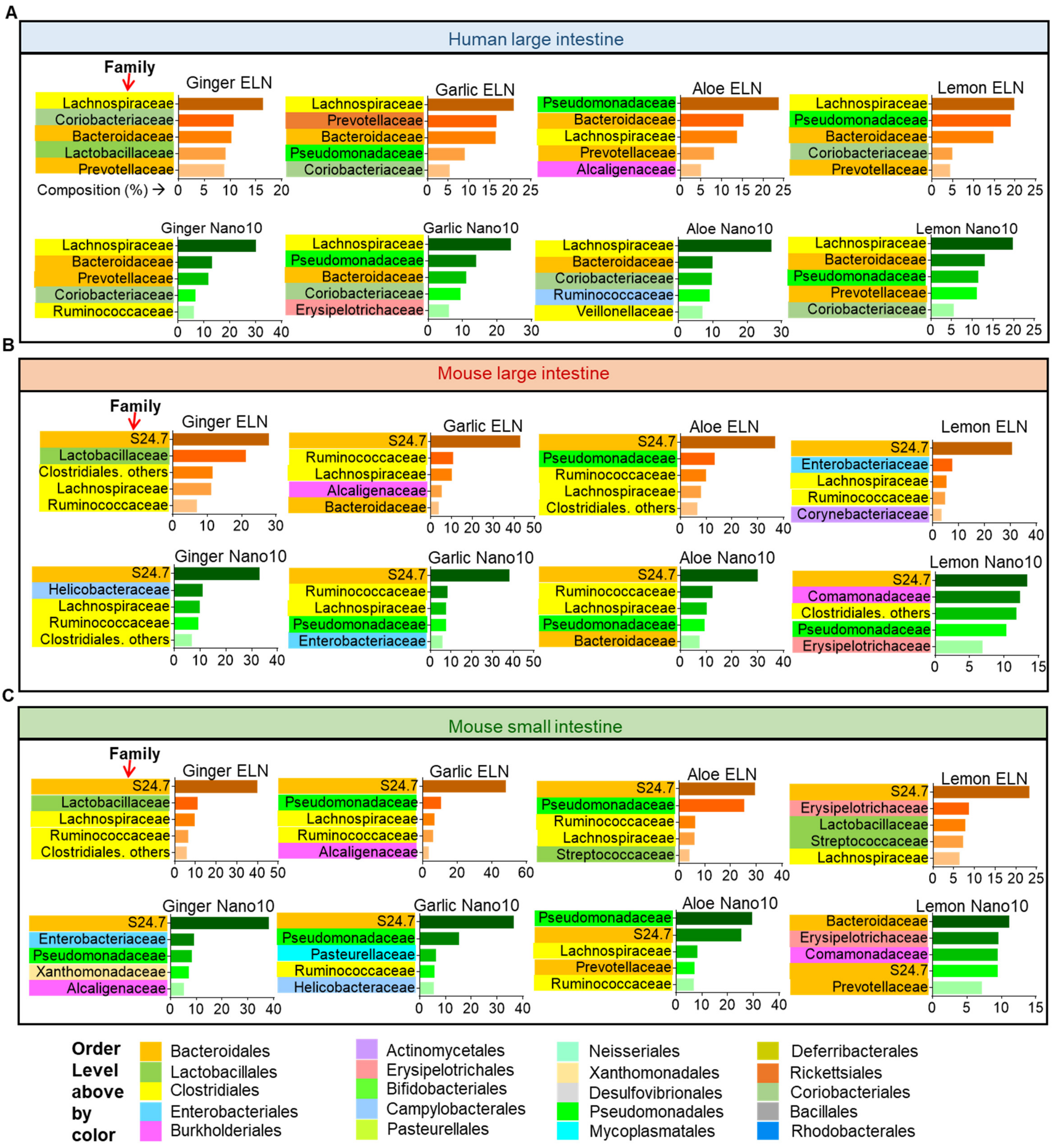

Supplementary Fig. 5 The top five bacterial families who referentially take up PNPs. A-C The PNP-PKH26^{+} bacterial from human feces **(A)**, hFB mouse LI **(B)** and SI **(C).** The DNA extracted for sequencing of 16S rRNA gene. Bar graphs indicate the average proportion of the top 5 PNP-PKH26^{+} bacteria at the family level using 16s rRNA gene sequencing. The background color represents the order of bacterial taxonomy. Source data are provided as a Source Data file.

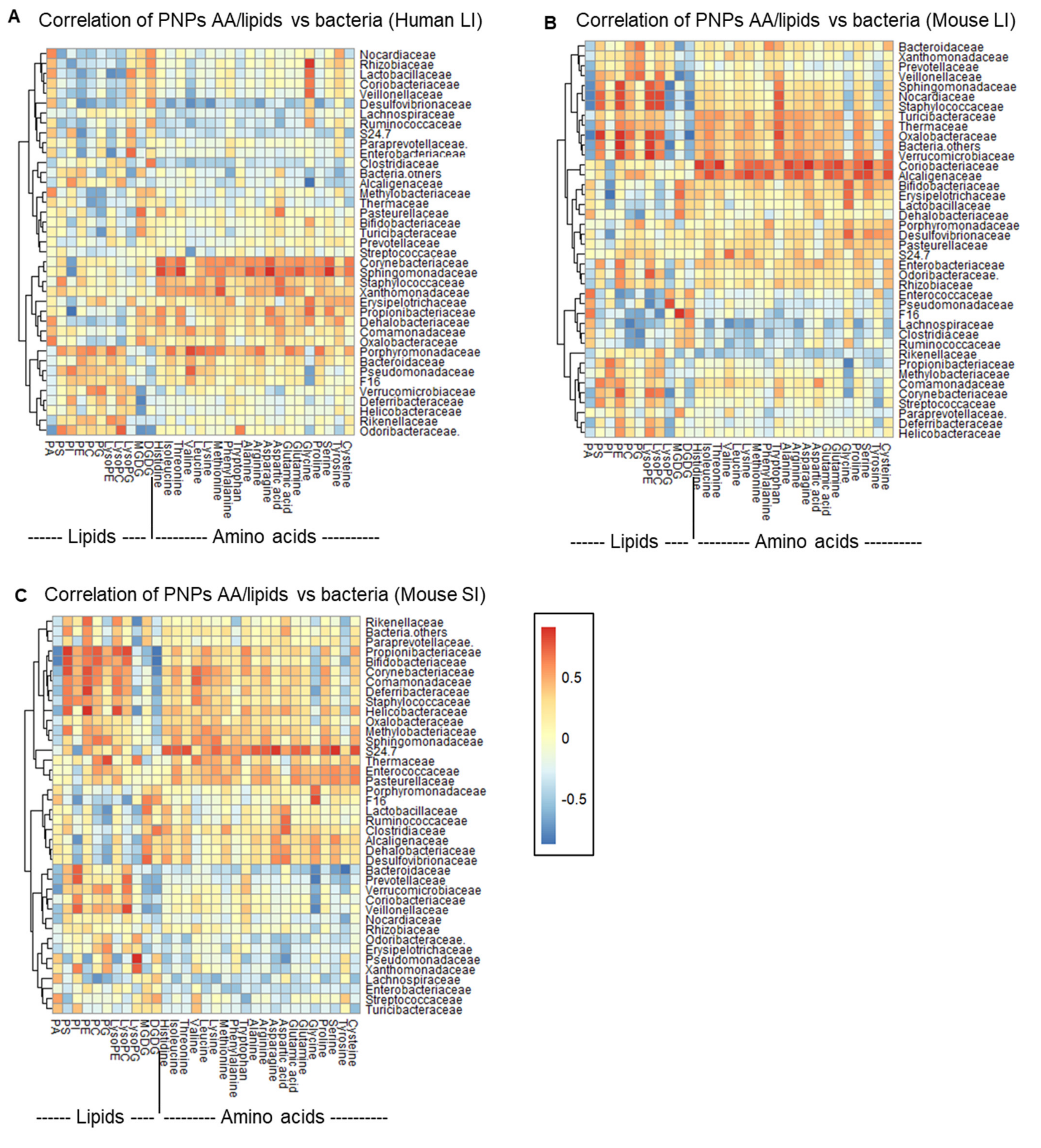

Supplementary Fig. 6 Bacteria uptake of PNPs is mediated by PNP lipids and amino acids (AA). A-C Heatmap indicating Spearman's correlation coefficients between the levels of PNP lipid or AA and the PNPs recipient bacteria in Human LI **(A)**, Mouse LI **(B)** and Mouse SI **(C)**, respectively. Source data are provided as a Source Data file.

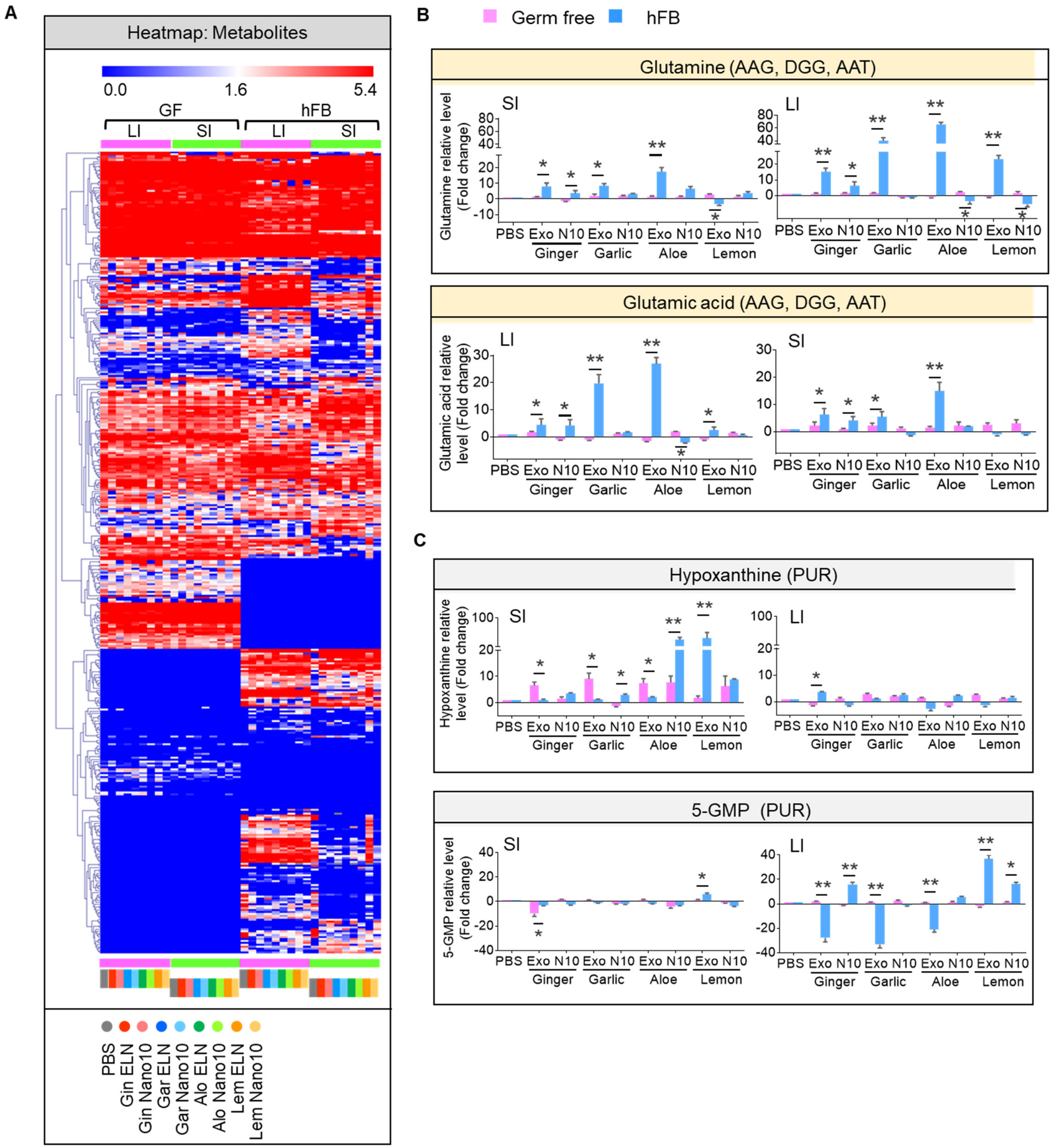

Supplementary Fig. 7 LC-MS analysis of gut bacterial metabolites. A Heatmap of gut metabolomics analysis in gut feces from GF mice and hFB mice treated with PNPs (0.5 g/kg, body weight) every other day for two months. **B-C** To verify the results of high-throughput LC-MS analysis, select metabolites included glutamine, glutamic acid (B), hypoxanthine and 5-GMP level (C) in gut fecal supernatant estimated with HPLC. Data are representative of three independent experiments (mean ± SD). *p < 0.05; **p < 0.01 (two-tailed t-test). Source data are provided as a Source Data file.

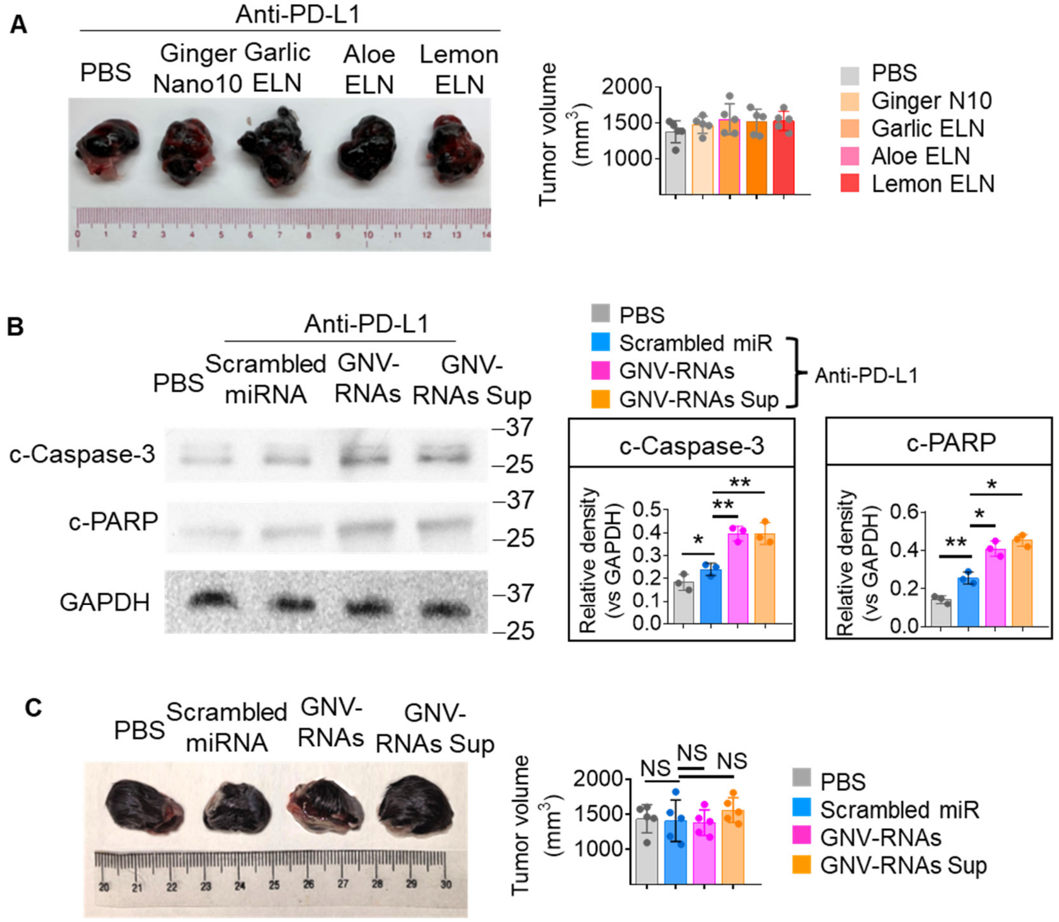

Supplementary Fig. 8 Ginger-nanovesicle encapsulated GELN-derived RNAs inhibits the growth and lung metastasis of B16F10 melanoma. A Representative B16F10 melanoma primary tumor (left panel) from tumor bearing hFB mice at 28 d post-injection of B16F10 cells along with anti-PD-L1 antibody with ginger-Nano10, garlic-ELN, aloe-ELN and lemon-ELN. **B** Western blot analysis of cleaved (c)-caspase-3 and c-PARP in melanoma tissues of mice treated with anti-PD-L1 described in Fig. 6C (left panel). The size (kDa) of protein MW indicated. Quantification of band intensity in western blot (right panel). **C** Representative B16F10 melanoma primary tumor (left panel) from tumor bearing hFB mice at 28 d post-injection of B16F10 cells along with GNV-RNAs or gut metabolites from GNV-RNAs treated mice. Quantification of primary tumor volume and metastasis nodule number (> 1 μm) (right panel). Data are representative of three independent experiments (mean ± SD). *$p < 0.05$; **$p < 0.01$ (two-tailed t-test). Source data are provided as a Source Data file.

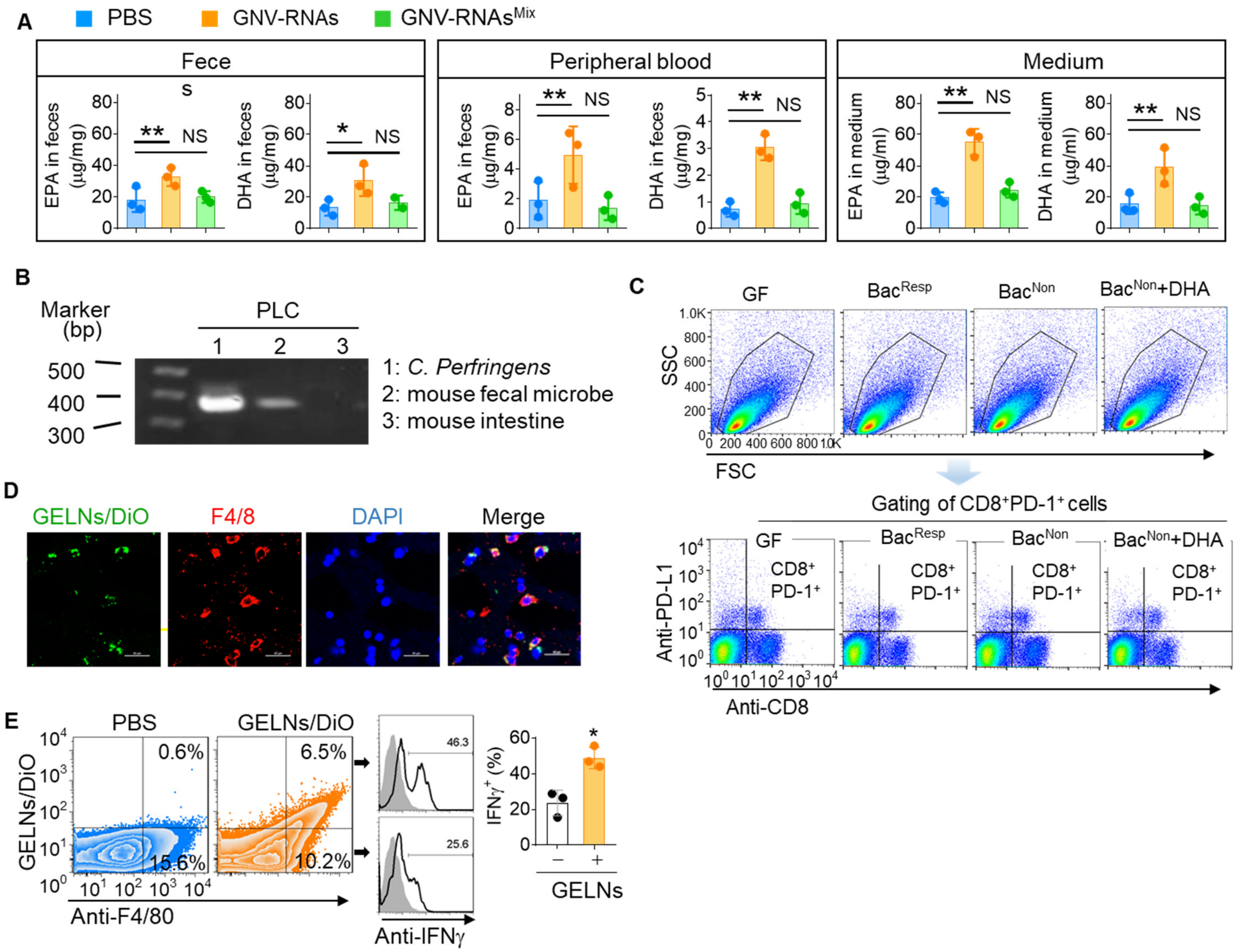

Supplementary Fig. 9 GELN-derived RNAs induce DHA and EPA in gut bacteria. A GNV-RNAs and mixtures of garlic, aloe and lemon derived NV-RNAs administered to hFB mice via oral gavage and incubated with *C. perf* in the growth medium. The feces, peripheral blood and medium collected from hFB mice and *C. perf*, respectively. The level of EPA and DHA estimated using HPLC analysis. $*p < 0.05$; $**p < 0.01$ (two-tailed t-test). **B** Representative immunoblot analysis shows PLC expression in *C. perf* and the gut feces of hFB but not the intestine of mice. **C** Monocytes isolated from the melanoma tissue in B16F10 tumor bearing mice generated in Fig. 9I. For FACS analysis, FSC vs SSC gating used to identify cells of interest while exclude debris and dead cells (top panel), followed by gating of $CD8^+PD\text{-}1^+$ population (bottom panel) for IFNγ analysis indicated in Fig. 9K. **D** C57BL/6 mice inoculated with B16F10 melanoma cell ($1x10^5$, n=5) and oral administered with GELNs labeled with green fluorescence dye DiO 0.5 g/kg (body weight, n=6). The monocytes were isolated from tumor using centrifugation on Percoll®. Representative immunofluorescence of the monocytes from tumor. Visualization of $F4/80^+$ and $GELNDiO^+$ cells by confocal microscopy. Scale bars, 20 µm. **E** Frequency of $IFN\gamma^+F4/80^+$ cells in DiO negative and DiO positive GELNs in tumor-infiltrating monocytes assessed using flow cytometry. Quantification of $IFN\gamma^+F4/80^+$ cells in DiO negative and DiO positive macrophages, respectively (right bar graph). Data are representative of three independent experiments (mean ± SD). $*p < 0.05$; $**p < 0.01$ (Chi-Square test). Source data are provided as a Source Data file.

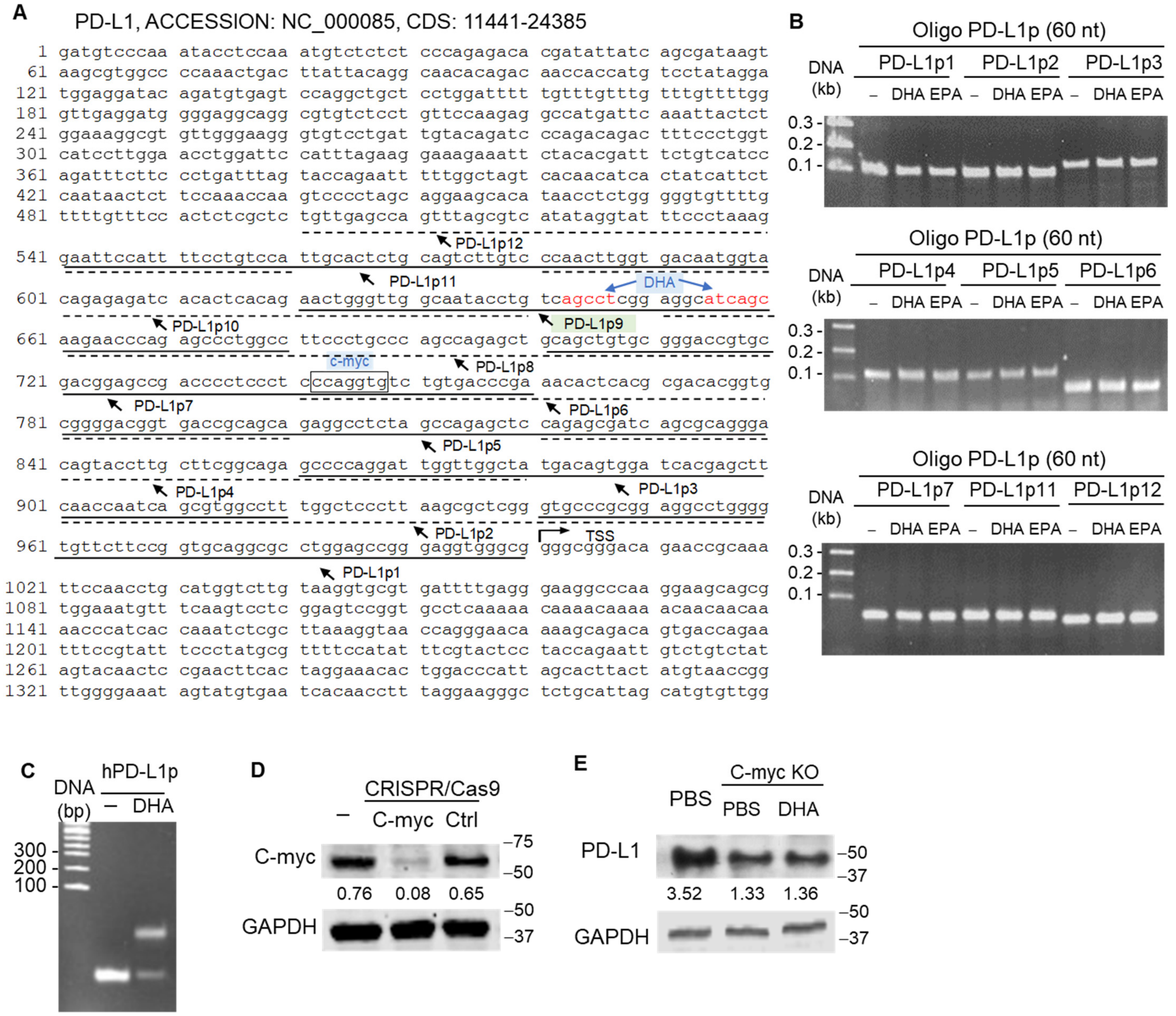

Supplementary Fig. 10 Identification of DHA binding motif on the promoter region of PD-L1. A Sequence of mouse PD-L1 (NC_000085, CDS: 11441-24385) promoter; Transcription start-site (TSS) marked by a bent arrow. The sequences of synthetic oligos are underlined and the names are indicated below the sequence. The sequences of the DHA binding motif are in red. **B** 10 pmol of synthetic DNA oligos PD-L1p1-7 and PD-L1p11-12 (bottom panel) (60 mer/each) correspond to the sequence on the promoter of PD-L1 incubated with DHA (1 μM) and EPA (1 μM) for 30 min at 37°C. The oligos separated on 15% PAGE and visualized with ethidium bromide. **C** The synthetic DNA oligo hPD-L1p correspond to the sequence of human PD-L1 promoter. Representative 15% PAGE for the oligo with or without DHA. **D** B16F10 cells transfected with c-myc CRISPR/Cas9 plasmid for 48 h and western blot analysis of c-myc expression. The size (kDa) of protein MW indicated. Numbers below western blots represent densitometry values normalized to the loading control. **E** Western blot analysis for PD-L1 expression in c-myc KO B16F10 cells treated with DHA. The size (kDa) of protein MW indicated. Numbers below western blots represent densitometry values normalized to the loading control. Data are representative of three independent experiments. Source data are provided as a Source Data file.

Supplementary Table 1 List of human gut microbiota composition evaluated by 16S rRNA sequencing

Taxonomy	Bacteria composition (%)														
	1	2	3	4	5	6	7	8	9	10	11	12	13	14	15
Bacteria.others	4.55	5.07	6.89	5.47	8.60	8.99	6.84	7.43	3.87	5.74	5.90	5.19	2.14	0.65	4.27
Corynebacteriaceae	0.00	0.00	0.00	0.00	0.00	0.00	0.00	0.00	0.00	0.00	0.00	0.00	0.00	0.00	0.01
Nocardiaceae	0.00	0.00	0.00	0.00	0.00	0.00	0.00	0.00	0.00	0.04	0.01	0.02	0.01	0.00	0.02
Propionibacteriaceae	0.00	0.00	0.00	0.00	0.00	0.00	0.00	0.00	0.00	0.00	0.00	0.00	0.00	0.00	0.00
Bifidobacteriaceae	0.00	0.00	0.00	0.00	0.01	0.00	0.00	0.03	0.04	0.02	0.00	0.05	0.23	1.42	0.16
Coriobacteriaceae	0.00	0.01	0.00	0.00	0.23	0.00	0.00	0.00	2.39	2.87	2.37	0.08	0.18	0.47	4.23
Bacteroidaceae	13.40	14.78	1.04	0.98	8.96	9.63	1.30	0.72	1.22	0.33	0.23	2.46	0.00	3.51	4.08
Porphyromonadaceae	1.86	1.51	1.39	1.10	2.48	3.82	1.15	1.30	5.60	4.34	0.91	1.65	16.46	8.27	4.86
Prevotellaceae	5.40	6.72	1.54	2.18	15.26	8.92	1.22	1.32	1.77	3.35	0.66	12.49	0.00	2.72	4.21
Rikenellaceae	4.17	3.37	2.27	1.69	5.54	5.39	2.10	3.00	4.07	5.71	1.27	0.42	0.00	2.98	14.46
S24.7	31.43	26.99	38.89	36.85	14.21	14.70	26.98	23.09	11.86	9.67	24.54	26.04	34.51	30.89	10.50
Odoribacteraceae.	0.21	0.21	1.50	1.41	0.82	0.62	1.51	1.63	0.00	0.00	0.00	0.00	0.00	0.00	0.00
Paraprevotellaceae.	2.61	2.61	0.52	0.72	1.72	2.41	0.43	0.42	0.00	0.00	0.00	0.00	0.00	0.00	0.00
Deferribacteraceae	3.09	2.50	3.75	3.17	2.33	3.28	3.27	2.30	0.53	2.40	0.66	1.03	0.00	0.41	0.98
Staphylococcaceae	0.00	0.00	0.00	0.00	0.00	0.00	0.00	0.00	0.00	0.00	0.00	0.00	0.00	0.00	0.00
Enterococcaceae	0.00	0.00	0.00	0.00	0.00	0.00	0.00	0.00	0.01	0.00	0.01	0.00	0.00	0.00	0.00
Lactobacillaceae	0.00	0.00	0.00	0.00	0.03	0.07	0.00	0.03	0.14	0.00	0.25	0.02	0.00	0.02	0.00
Streptococcaceae	0.00	0.00	0.00	0.00	0.00	0.00	0.00	0.00	0.01	0.28	0.89	0.78	0.11	0.01	0.02
Clostridiales. others	7.79	7.68	20.02	22.35	11.17	14.35	25.10	25.42	30.41	22.50	26.45	19.66	20.97	13.44	19.55
Dehalobacteriaceae	0.23	0.23	0.31	0.31	0.55	0.81	0.24	0.15	0.00	0.00	0.00	0.00	0.53	0.04	0.51
Lachnospiraceae	3.64	3.88	7.39	7.43	6.96	3.41	8.92	10.13	10.46	15.14	12.31	7.60	5.33	12.37	7.93
Ruminococcaceae	7.30	6.69	6.84	6.24	8.66	10.40	11.52	11.77	22.18	21.86	20.47	15.11	12.08	11.81	18.19
Veillonellaceae	0.00	0.00	0.00	0.03	1.13	0.00	0.03	0.00	0.39	1.40	0.00	3.78	0.35	5.37	2.58
Erysipelotrichaceae	0.46	0.45	0.45	0.54	0.27	0.33	2.15	2.29	0.31	0.05	0.63	2.22	0.44	0.15	0.84
Methylobacteriaceae	0.00	0.00	0.00	0.00	0.00	0.00	0.00	0.00	0.00	0.00	0.00	0.00	0.00	0.00	0.00
Rhizobiaceae	0.00	0.00	0.00	0.00	0.00	0.00	0.00	0.00	0.00	0.00	0.00	0.00	0.00	0.00	0.00
Sphingomonadaceae	0.00	0.00	0.01	0.00	0.00	0.00	0.00	0.00	0.00	0.00	0.00	0.00	0.00	0.00	0.00
Alcaligenaceae	4.91	8.51	2.37	3.08	3.45	3.92	2.00	4.00	0.37	0.86	0.95	0.29	0.85	0.84	0.79
Comamonadaceae	0.00	0.00	0.00	0.00	0.00	0.00	0.00	0.00	0.00	0.00	0.00	0.00	0.00	0.00	0.00
Oxalobacteraceae	0.00	0.00	0.00	0.00	0.00	0.00	0.00	0.00	0.01	0.00	0.00	0.01	0.00	0.00	0.13
Desulfovibrionaceae	1.65	1.72	1.48	1.41	1.09	1.41	0.85	0.61	0.52	0.34	0.42	0.19	0.00	0.30	0.29
Helicobacteraceae	4.08	4.59	2.93	4.54	3.73	4.89	3.20	3.53	2.32	2.28	0.58	0.07	1.53	0.12	0.22
Enterobacteriaceae	2.66	1.96	0.00	0.00	1.22	1.02	0.09	0.03	0.35	0.53	0.22	0.02	1.62	3.52	0.14
Pasteurellaceae	0.00	0.00	0.00	0.00	0.55	0.65	0.00	0.00	0.00	0.00	0.00	0.00	0.00	0.00	0.00
Pseudomonadaceae	0.00	0.00	0.01	0.00	0.10	0.00	0.00	0.00	0.01	0.06	0.00	0.64	0.01	0.01	0.00
Xanthomonadaceae	0.00	0.00	0.00	0.00	0.00	0.00	0.00	0.00	0.00	0.00	0.00	0.00	0.00	0.00	0.00
F16	0.39	0.44	0.40	0.50	0.76	0.98	1.10	0.80	1.14	0.05	0.28	0.19	2.66	0.46	1.00
Verrucomicrobiaceae	0.17	0.08	0.00	0.00	0.17	0.00	0.00	0.00	0.00	0.17	0.00	0.00	0.00	0.21	0.00
Thermaceae	0.00	0.00	0.00	0.00	0.00	0.00	0.00	0.00	0.00	0.00	0.00	0.00	0.00	0.00	0.00

Supplementary Table 2 List of mouse gut microbiota composition evaluated by 16S rRNA sequencing

Taxonomy	Bacteria composition (%)													
	Large intestine							Small intestine						
	1	2	3	4	5	6	7	1	2	3	4	5	6	7
Bacteria.others	2.86	2.59	3.00	3.20	3.68	3.02	3.60	2.56	4.19	7.39	4.69	6.04	4.70	2.45
Corynebacteriaceae	0.00	0.00	0.00	0.00	0.03	0.00	0.00	0.00	0.00	0.16	0.00	0.00	0.00	0.09
Nocardiaceae	0.00	0.00	0.00	0.00	0.00	0.00	0.00	0.00	0.00	0.00	0.00	0.00	0.00	0.00
Propionibacteriaceae	0.00	0.00	0.00	0.00	0.05	0.00	0.00	0.00	0.00	0.00	0.00	0.00	0.00	0.09
Bifidobacteriaceae	0.00	0.00	0.00	0.00	0.00	0.00	0.00	0.00	0.00	0.00	0.00	0.00	0.00	0.00
Coriobacteriaceae	0.00	0.00	0.00	0.00	0.00	0.00	0.00	0.00	0.00	0.00	0.00	0.06	0.00	0.00
Bacteroidaceae	1.85	1.52	0.20	0.50	1.80	7.34	0.70	4.32	13.00	2.36	0.12	0.23	0.54	0.17
Porphyromonadaceae	0.46	0.39	0.20	0.00	0.32	0.97	0.20	2.04	1.82	1.00	0.00	0.00	0.01	2.10
Prevotellaceae	2.62	2.19	1.10	1.60	0.00	10.48	3.40	0.00	0.00	0.68	0.86	0.00	1.80	1.86
Rikenellaceae	6.42	5.66	3.70	3.40	6.12	6.59	1.80	15.72	14.82	8.48	1.34	1.86	3.36	5.18
S24.7	26.00	28.23	26.40	27.30	40.26	23.98	48.40	24.72	27.43	58.60	74.61	74.39	58.34	40.59
Odoribacteraceae.	4.53	5.48	0.90	0.90	5.02	1.62	0.80	6.00	6.89	5.07	0.50	0.94	2.41	4.88
Paraprevotellaceae.	0.52	0.37	0.10	0.20	0.62	2.38	0.20	0.00	0.00	0.79	0.00	0.00	0.00	1.11
Deferribacteraceae	1.77	3.60	1.50	1.40	5.78	1.94	0.60	0.12	0.39	1.69	1.60	2.53	3.73	5.45
Staphylococcaceae	0.00	0.34	0.00	0.00	0.08	0.00	0.00	0.00	0.00	0.00	0.00	0.00	0.04	0.00
Enterococcaceae	0.00	0.00	0.00	0.00	0.00	0.00	0.00	0.00	0.00	0.00	0.00	0.00	0.00	0.00
Lactobacillaceae	0.03	0.04	0.10	0.00	0.13	0.43	0.00	0.24	0.00	0.95	0.82	5.80	1.19	2.61
Streptococcaceae	0.00	0.00	0.00	0.00	0.00	0.00	0.00	0.36	0.26	0.00	0.00	0.31	0.00	2.97
Clostridiales. others	26.78	32.77	48.10	45.40	21.99	26.46	31.90	24.24	19.11	8.54	14.02	3.58	16.35	19.25
Dehalobacteriaceae	0.00	0.00	0.00	0.20	0.00	0.00	0.10	0.00	0.13	0.15	0.00	2.47	0.15	2.88
Lachnospiraceae	1.07	0.97	4.10	3.40	0.62	2.16	2.70	2.52	0.91	0.28	0.06	0.00	0.44	0.28
Ruminococcaceae	0.00	0.00	7.20	9.30	0.00	7.13	4.00	13.56	4.81	0.00	0.00	0.00	0.00	0.74
Veillonellaceae	25.10	15.84	0.00	0.00	13.50	0.00	0.00	0.00	0.00	3.85	0.86	1.80	6.96	6.55
Erysipelotrichaceae	0.00	0.00	0.00	0.00	0.00	0.00	0.00	1.44	3.38	0.00	0.00	0.00	0.00	0.61
Methylobacteriaceae	0.00	0.00	0.00	0.00	0.00	0.00	0.00	0.00	0.00	0.00	0.00	0.00	0.00	0.00
Rhizobiaceae	0.00	0.00	0.00	0.00	0.00	0.00	0.00	0.00	0.00	0.00	0.00	0.00	0.00	0.00
Sphingomonadaceae	0.00	0.00	0.00	0.00	0.00	0.00	0.00	0.00	0.00	0.00	0.00	0.00	0.00	0.00
Alcaligenaceae	0.00	0.00	0.10	0.10	0.00	3.02	0.40	0.00	0.00	0.00	0.50	0.00	0.00	0.00
Comamonadaceae	0.00	0.00	0.00	0.00	0.00	0.00	0.00	0.00	0.00	0.00	0.00	0.00	0.00	0.00
Oxalobacteraceae	0.00	0.00	0.00	0.00	0.00	0.00	0.00	0.00	0.00	0.00	0.00	0.00	0.00	0.01
Desulfovibrionaceae	0.00	0.00	0.70	0.50	0.00	2.38	0.50	1.32	1.56	0.00	0.00	0.00	0.00	0.00
Helicobacteraceae	0.00	0.00	2.30	2.40	0.00	0.00	0.70	0.60	0.52	0.00	0.00	0.00	0.00	0.13
Enterobacteriaceae	0.00	0.00	0.00	0.00	0.00	0.00	0.00	0.12	0.00	0.00	0.00	0.00	0.00	0.00
Pasteurellaceae	0.00	0.00	0.00	0.00	0.00	0.00	0.00	0.00	0.00	0.00	0.00	0.00	0.00	0.00
Pseudomonadaceae	0.00	0.00	0.00	0.00	0.00	0.00	0.00	0.00	0.00	0.00	0.00	0.00	0.00	0.00
Xanthomonadaceae	0.00	0.00	0.00	0.00	0.00	0.00	0.00	0.00	0.00	0.00	0.00	0.00	0.00	0.00
F16	0.00	0.00	0.30	0.20	0.00	0.11	0.00	0.12	0.78	0.00	0.00	0.00	0.00	0.00
Verrucomicrobiaceae	0.00	0.00	0.00	0.00	0.00	0.00	0.00	0.00	0.00	0.00	0.00	0.00	0.00	0.00
Thermaceae	0.00	0.00	0.00	0.00	0.00	0.00	0.00	0.00	0.00	0.00	0.00	0.00	0.00	0.00

Supplementary Table 3 List of PNPs taking up human gut microbiota composition *in vitro* evaluated by 16S rRNA sequencing

Taxonomy	PNPs take up bacteria composition (%)							
	Ginger		Garlic		Aloe		Lemon	
	ELN	Nano10	ELN	Nano10	ELN	Nano10	ELN	Nano10
Bacteria.others	5.75	6.64	3.16	5.68	7.74	3.33	8.57	10.72
Corynebacteriaceae	0.56	0.02	0.23	0.18	0.01	0.02	0.69	0.00
Nocardiaceae	0.03	0.02	0.03	0.00	0.12	0.01	0.01	0.01
Propionibacteriaceae	1.42	0.23	0.22	0.19	0.18	0.18	0.29	0.07
Bifidobacteriaceae	2.02	2.48	0.57	2.43	0.43	0.92	2.19	1.64
Coriobacteriaceae	10.72	6.72	5.45	9.35	3.26	9.84	4.95	5.61
Bacteroidaceae	10.25	13.25	16.45	11.08	15.24	9.99	14.77	13.03
Porphyromonadaceae	1.53	1.27	1.73	2.25	1.68	0.72	1.55	2.17
Prevotellaceae	8.88	11.64	16.71	0.78	8.16	5.02	4.51	11.20
Rikenellaceae	0.37	0.77	0.75	1.01	0.56	0.66	0.58	1.36
S24.7	0.80	0.27	0.62	0.39	0.92	4.16	0.11	0.68
Odoribacteraceae.	0.56	0.48	0.62	0.53	0.50	0.65	0.64	1.07
Paraprevotellaceae.	0.09	0.02	0.05	0.03	0.05	0.14	0.05	0.05
Deferribacteraceae	0.12	0.04	0.22	0.05	0.14	0.44	0.05	0.16
Staphylococcaceae	0.10	0.01	0.00	0.38	0.00	0.00	0.29	0.04
Enterococcaceae	0.00	0.00	0.00	0.00	0.00	0.00	0.00	0.00
Lactobacillaceae	9.10	0.58	0.39	1.19	0.46	1.85	0.57	0.19
Streptococcaceae	5.52	0.43	0.16	0.36	0.11	0.76	0.70	0.50
Turicibacteraceae	0.03	0.11	0.00	0.01	0.00	0.00	0.01	0.03
Clostridiales. others	2.26	5.17	2.97	2.36	1.93	3.03	2.40	3.44
Dehalobacteriaceae	0.01	0.00	0.00	0.00	0.00	0.00	0.00	0.00
Lachnospiraceae	16.35	30.06	20.89	24.09	13.80	27.02	19.82	19.81
Ruminococcaceae	4.28	6.06	5.22	4.18	3.76	9.22	2.64	4.01
Veillonellaceae	4.89	4.69	3.87	4.61	2.43	7.04	3.86	3.30
Erysipelotrichaceae	2.98	3.45	4.83	6.11	1.89	3.45	2.91	0.91
Methylobacteriaceae	0.08	0.01	0.00	0.01	0.02	0.01	0.00	0.05
Rhizobiaceae	0.83	1.20	0.31	0.68	0.17	0.35	0.19	0.23
Sphingomonadaceae	0.40	0.08	0.09	0.06	0.04	0.03	0.09	0.04
Alcaligenaceae	0.19	0.44	0.60	0.13	5.01	0.50	3.88	4.20
Comamonadaceae	0.61	0.05	0.08	0.28	0.63	0.03	0.08	0.16
Oxalobacteraceae	1.46	0.07	0.00	0.12	0.09	0.01	0.02	0.08
Desulfovibrionaceae	0.40	0.34	0.15	0.28	0.18	0.46	0.15	0.31
Helicobacteraceae	0.07	0.04	0.10	0.05	0.06	0.73	0.02	0.08
Enterobacteriaceae	3.49	2.36	2.86	2.70	4.69	5.57	2.78	1.36
Pasteurellaceae	0.02	0.01	0.00	0.00	0.01	0.00	0.06	0.01
Pseudomonadaceae	1.52	0.28	9.10	13.89	23.77	2.26	19.05	11.45
Xanthomonadaceae	1.42	0.04	0.27	2.58	1.23	0.03	1.08	1.33
F16	0.00	0.00	0.02	0.00	0.04	0.00	0.00	0.00
Verrucomicrobiaceae	0.22	0.68	1.31	2.04	0.58	1.60	0.47	0.69
Thermaceae	0.71	0.02	0.00	0.00	0.18	0.02	0.03	0.05

Each group was pooled from three independent samples.

Supplementary Table 4 List of PNPs taking up bacteria composition in large intestine of mice evaluated by 16S rRNA sequencing

Taxonomy	PNPs take up bacteria composition (%)							
	Ginger		Garlic		Aloe		Lemon	
	ELN	Nano10	ELN	Nano10	ELN	Nano10	ELN	Nano10
Bacteria.others	1.62	3.18	5.03	3.15	1.33	0.57	6.04	5.58
Corynebacteriaceae	0.00	0.01	0.07	0.00	0.07	0.00	0.17	2.86
Nocardiaceae	0.00	0.00	0.15	0.00	0.00	0.00	0.83	3.36
Propionibacteriaceae	0.00	0.00	0.00	0.00	0.05	0.00	0.02	1.44
Bifidobacteriaceae	0.26	0.42	0.12	0.40	0.04	0.00	0.06	0.03
Coriobacteriaceae	1.45	0.28	0.95	0.42	0.05	0.01	0.69	0.05
Bacteroidaceae	1.73	1.61	6.40	3.40	1.46	12.37	5.15	1.68
Porphyromonadaceae	1.02	1.51	3.39	2.34	0.04	2.66	0.58	0.15
Prevotellaceae	0.47	1.93	2.42	2.94	1.00	10.98	4.53	1.86
Rikenellaceae	0.29	1.37	1.16	1.09	0.66	2.06	0.95	1.56
S24.7	23.87	28.78	56.62	35.91	39.10	23.19	25.16	26.80
Odoribacteraceae.	0.06	0.43	0.28	0.18	0.03	0.05	0.12	1.20
Paraprevotellaceae.	0.62	1.35	0.64	0.74	3.58	0.22	1.15	0.86
Deferribacteraceae	0.35	2.12	0.66	0.39	0.18	0.50	2.07	0.78
Staphylococcaceae	0.00	0.00	0.04	0.00	0.00	0.00	0.09	0.56
Enterococcaceae	0.08	0.00	0.00	0.00	0.07	0.05	0.00	0.00
Lactobacillaceae	14.35	6.58	0.05	0.36	0.00	0.10	0.43	0.07
Streptococcaceae	0.01	0.05	0.00	0.17	0.00	0.02	0.46	0.62
Turicibacteraceae	0.01	0.00	0.32	0.00	0.01	0.00	0.09	0.01
Clostridiales. others	16.06	13.93	0.73	2.74	13.05	5.38	2.16	23.56
Dehalobacteriaceae	0.32	0.13	0.03	0.11	0.13	0.00	0.08	0.20
Lachnospiraceae	16.32	10.95	3.07	3.32	6.53	12.24	5.64	7.95
Ruminococcaceae	8.01	7.24	1.16	4.60	2.45	6.26	3.76	7.07
Veillonellaceae	0.01	0.00	0.22	0.07	0.01	1.44	0.80	0.07
Erysipelotrichaceae	1.20	1.22	0.76	0.65	0.10	0.02	0.90	0.02
Methylobacteriaceae	0.00	0.00	0.02	0.00	0.03	0.00	0.00	2.29
Rhizobiaceae	0.00	0.01	0.05	0.00	0.00	0.00	1.46	0.00
Sphingomonadaceae	0.00	0.01	0.15	0.00	0.00	0.00	2.42	1.07
Alcaligenaceae	3.15	2.12	8.72	6.93	1.03	0.85	3.39	0.58
Comamonadaceae	0.01	0.00	0.00	0.03	0.04	0.00	0.71	0.38
Oxalobacteraceae	0.00	0.00	0.12	0.01	0.00	0.00	1.12	0.45
Desulfovibrionaceae	1.45	2.06	3.27	2.03	0.58	0.92	0.60	0.51
Helicobacteraceae	0.43	3.47	1.92	0.91	0.96	0.87	1.59	1.61
Enterobacteriaceae	2.21	7.63	0.54	11.89	0.05	0.04	13.70	4.46
Pasteurellaceae	0.00	0.01	0.01	0.15	0.00	0.00	0.00	0.00
Pseudomonadaceae	4.44	1.14	0.58	14.87	26.74	19.08	1.35	0.22
Xanthomonadaceae	0.00	0.00	0.24	0.06	0.41	0.10	6.81	0.00
F16	0.25	0.50	0.01	0.17	0.30	0.08	0.14	0.12
Verrucomicrobiaceae	0.02	0.02	0.09	0.02	0.00	0.00	0.03	0.04
Thermaceae	0.00	0.00	0.10	0.00	0.00	0.00	4.89	0.00

Each group was pooled from three independent samples.

Supplementary Table 5 List of PNPs taking up bacteria composition in small intestine of mice evaluated by 16S rRNA sequencing

Taxonomy	PNPs take up bacteria composition (%)							
	Ginger		Garlic		Aloe		Lemon	
	ELN	Nano10	ELN	Nano10	ELN	Nano10	ELN	Nano10
Bacteria.others	1.43	1.80	0.52	4.04	1.07	0.90	5.59	3.73
Corynebacteriaceae	0.00	0.00	0.03	0.50	0.06	0.00	3.52	0.24
Nocardiaceae	0.00	0.00	0.00	0.00	0.00	0.01	0.43	0.00
Propionibacteriaceae	0.00	0.00	0.03	0.57	0.00	0.02	0.31	2.34
Bifidobacteriaceae	0.00	0.01	0.09	0.24	0.04	0.17	2.43	0.22
Coriobacteriaceae	0.11	0.04	1.15	0.04	1.96	0.40	0.24	0.91
Bacteroidaceae	3.07	3.46	1.46	3.65	5.67	6.92	6.98	22.39
Porphyromonadaceae	1.85	3.77	0.88	3.60	0.17	0.83	0.49	1.72
Prevotellaceae	1.40	0.36	1.55	1.04	4.91	13.04	5.99	14.46
Rikenellaceae	0.68	1.65	0.35	0.71	0.71	0.21	1.77	2.85
S24.7	25.67	24.49	47.32	22.16	9.50	1.57	31.10	13.17
Odoribacteraceae.	0.02	0.07	0.03	0.24	0.07	0.25	0.16	0.03
Paraprevotellaceae.	0.24	2.06	0.15	0.59	0.01	0.06	1.69	0.63
Deferribacteraceae	0.19	0.30	0.38	0.39	0.50	0.06	3.23	2.12
Staphylococcaceae	0.00	0.00	0.00	1.30	0.07	0.00	0.92	0.97
Enterococcaceae	0.00	0.08	2.03	0.26	0.00	0.00	0.00	0.00
Lactobacillaceae	11.02	2.76	0.13	0.02	0.27	0.04	7.13	0.47
Streptococcaceae	0.01	0.58	0.17	0.57	8.18	0.01	0.00	0.04
Turicibacteraceae	0.00	0.00	0.00	0.00	0.70	0.00	0.00	0.00
Clostridiales. others	11.30	2.75	0.91	4.02	1.08	1.86	3.95	3.84
Dehalobacteriaceae	1.65	0.22	0.05	0.11	0.00	0.00	0.08	0.14
Lachnospiraceae	13.67	4.78	1.89	2.92	5.06	7.78	2.04	10.76
Ruminococcaceae	9.83	3.48	1.86	3.42	2.14	2.46	6.94	7.49
Veillonellaceae	0.01	0.00	0.97	0.04	0.83	0.80	0.55	1.90
Erysipelotrichaceae	0.12	0.45	1.49	0.43	0.82	1.50	1.41	0.40
Methylobacteriaceae	0.00	0.00	0.00	0.09	0.00	0.00	1.93	0.00
Rhizobiaceae	0.00	0.00	0.00	0.00	0.04	0.00	0.61	0.00
Sphingomonadaceae	0.00	0.01	0.30	0.05	0.03	0.01	0.67	0.00
Alcaligenaceae	4.54	8.58	4.39	3.06	1.10	0.35	3.48	3.70
Comamonadaceae	0.00	0.01	0.01	1.78	0.04	0.00	0.18	0.67
Oxalobacteraceae	0.00	0.00	0.06	0.00	0.07	0.00	0.23	0.00
Desulfovibrionaceae	2.85	2.77	0.57	0.62	0.30	0.10	1.16	0.88
Helicobacteraceae	0.24	1.20	0.30	1.36	0.14	0.10	2.06	1.91
Enterobacteriaceae	0.24	17.48	1.92	0.35	2.49	1.32	2.42	1.71
Pasteurellaceae	0.01	0.53	7.34	12.73	0.00	0.00	0.00	0.01
Pseudomonadaceae	9.53	15.97	21.13	27.89	50.27	58.34	0.05	0.00
Xanthomonadaceae	0.05	0.00	0.31	0.64	1.58	0.66	0.00	0.27
F16	0.34	0.37	0.00	0.10	0.00	0.01	0.03	0.00
Verrucomicrobiaceae	0.00	0.04	0.22	0.04	0.13	0.30	0.30	0.11
Thermaceae	0.00	0.00	0.10	0.50	0.07	0.01	0.00	0.00

Each group was pooled from three independent samples.

Supplementary Table 6 Composition of lipids in PNPs using LC-MS (nmol/mg PNPs)

Name	Ginger				Garlic				Aloe				Lemon			
	ELN	ELN	Nano10	Nano10	ELN	ELN	Nano10	Nano10	ELN	ELN	Nano10	Nano10	ELN	ELN	Nano10	Nano10
Phosphatidic acid (PA)	41.52	35.42	18.37	12.58	7.19	3.82	8.40	11.93	33.60	35.28	9.34	7.25	1.93	5.01	2.54	6.74
Phosphatidylserine (PS)	0.17	0.08	0.02	0.06	0.80	0.35	0.58	1.25	0.04	0.08	0.04	0.12	4.21	2.36	0.71	1.51
Phosphatidylinositol (PI)	3.71	2.04	0.03	0.13	1.35	6.29	4.82	8.34	10.54	11.21	10.76	8.17	7.66	4.46	19.17	12.17
Phosphatidylethanolamine (PE)	0.57	0.10	0.33	1.55	20.92	11.20	12.06	6.89	0.28	0.39	0.13	0.12	29.95	37.11	35.50	25.50
Phosphatidylcholine (PC)	0.10	0.03	0.64	0.41	54.66	50.45	40.56	43.57	0.68	2.11	1.31	4.07	41.87	30.52	31.93	33.93
Phosphatidylglycerol (PG)	0.75	0.36	0.46	0.15	4.07	10.34	9.20	3.24	3.34	1.88	2.12	7.02	3.39	4.93	0.46	0.79
LysoPE	0.11	0.03	0.17	0.72	0.56	1.11	0.36	0.81	0.03	0.11	0.01	0.01	0.68	0.82	1.98	1.98
LysoPC	0.02	0.02	0.02	0.06	1.91	0.44	0.17	0.33	0.05	0.07	0.17	0.60	0.20	0.77	0.29	5.89
LysoPG	5.09	2.07	0.16	0.25	2.04	4.56	15.30	9.83	5.21	9.84	57.53	63.88	0.06	0.16	0.01	0.01
Monogalactosyldiacylglycerol (MGDG)	14.05	28.45	29.37	41.45	3.70	7.40	5.15	9.60	18.62	20.96	2.15	3.59	8.99	10.83	6.04	8.04
Digalactosyldiacylglycerol (DGDG)	33.90	31.43	50.46	42.63	2.82	4.04	3.43	4.19	27.61	18.08	16.45	5.16	1.08	3.03	1.42	3.42

Supplementary Table 7 Composition of amino acids in PNPs using LC-MS (intensity)

	Ginger				Garlic				Aloe				Lemon			
Name	ELN	ELN	Nano10	Nano10	ELN	ELN	Nano10	Nano10	ELN	ELN	Nano10	Nano10	ELN	ELN	Nano10	Nano10
Histidine	6.7E+08	5.8E+08	2.1E+07	2E+07	3.9E+08	3.2E+08	2.7E+08	2.2E+08	1.2E+08	9.6E+07	2860487	2346635	5.6E+08	4.2E+08	2.8E+08	2.1E+08
Isoleucine	1.9E+07	2.3E+07	858538	1086833	4.4E+07	4E+07	3.3E+07	2.2E+07	3562403	4547943	585582	678322	2.3E+07	2.7E+07	1.1E+07	8455902
Threonine	3E+07	2.3E+07	1213752	1312380	2.3E+07	1.6E+07	5613604	7435956	1278458	1446625	50352.2	44146.8	8494201	6868285	3118440	3857988
Valine	3.1E+08	2.5E+08	3.9E+07	3.2E+07	5.3E+08	4.3E+08	8.7E+08	7E+08	6.6E+08	4.1E+08	3.2E+07	3.4E+07	4.7E+08	3.4E+08	2.8E+08	3E+08
Leucine	5.7E+08	6.4E+08	4.8E+07	5.1E+07	1E+09	7.8E+08	1E+09	7.8E+08	2E+08	1.2E+08	6.3E+07	4.9E+07	7.1E+08	8.5E+08	1.9E+08	2.5E+08
Lysine	1.1E+08	1.4E+08	5739024	4859701	1.1E+09	1.5E+09	3.4E+08	4E+08	6.8E+07	5.2E+07	24714	1000	1.2E+08	1.5E+08	5.9E+07	4.9E+07
Methionine	1.3E+08	1.7E+08	1.1E+07	8835094	1.3E+08	1.6E+08	2.4E+08	2.9E+08	2.3E+07	3.1E+07	524708	480884	8.8E+07	1.1E+08	4.4E+07	3.9E+07
Phenylalanine	506422	388125	1000	2100	2.1E+07	1.8E+07	1E+07	7845972	1000	12471	15502	916.48	378889	328914	1000	6548
Tryptophan	2.3E+08	2.3E+08	1.1E+07	8739265	3.9E+08	3.2E+08	2.5E+08	1.8E+08	1.8E+07	1.4E+07	1.9E+07	1.3E+07	6.9E+08	6E+08	3.9E+08	3.2E+08
Alanine	3.7E+07	4.2E+07	1757649	1210850	5.6E+07	4E+07	3.6E+07	2.8E+07	3531662	3836698	268157	227431	1.5E+07	1.7E+07	5894941	4852707
Arginine	2.3E+09	1.8E+09	5.3E+07	4.1E+07	1.6E+09	1.4E+10	5.8E+09	4.7E+09	4.2E+08	4.7E+08	1.6E+07	2.2E+07	3E+09	1.9E+09	8.8E+08	1.2E+09
Asparagine	3.4E+08	4.2E+08	1.1E+07	1.3E+07	1.8E+08	1.2E+08	3.9E+07	3.6E+07	5714688	2487957	225661	170154	8.9E+07	6.1E+07	2.3E+07	3E+07
Aspartic acid	3.2E+08	3.3E+08	5546731	4283468	2.2E+07	3.3E+07	3.6E+07	3.2E+07	1.5E+07	1.1E+07	734849	573475	1.1E+08	8.3E+07	4E+07	3E+07
Glutamic acid	7E+07	5.9E+07	2423570	1821153	1.7E+08	1.2E+08	6.6E+07	7.1E+07	1.2E+07	1.5E+07	601922	368353	5.5E+07	5.9E+07	2.2E+07	2.3E+07
Glutamine	9.7E+07	1.1E+08	3046053	2291647	3.4E+08	2.7E+08	5.2E+07	5.5E+07	4496591	3021260	51070.2	36804.8	1.3E+07	1.8E+07	7787155	6403588
Glycine	6604605	5547988	2450201	2015561	1000	24816.5	32411	1100	1000	11000	24571	1000	12581	916.48	1000	3589
Proline	1.1E+09	9.8E+08	5.3E+07	4E+07	3.7E+09	2.7E+09	1.2E+09	1.8E+09	1.9E+08	1.5E+08	1.6E+07	1.2E+07	1.7E+09	1.3E+09	5.8E+08	3.3E+08
Serine	3.4E+08	2.9E+08	1.4E+07	1.7E+07	1.4E+08	9.9E+08	4.5E+07	6.3E+07	9464476	6674003	485233	789706	4E+07	4.6E+07	1.9E+07	1.2E+07
Tyrosine	3.9E+08	4.2E+08	9360332	6878557	4E+08	2.9E+08	3.4E+08	2.2E+08	6E+07	3.5E+07	7565771	5284214	2077979	2271018	1992927	2826477
Cysteine	1000	22116.5	13381	1000	3.6E+07	4.2E+07	3633875	5330374	1000	916.48	1000	12544	35448	1000	15428	916.48

Supplementary Table 8 List of significant correlation (p<0.05) between PNP lipids or amino acids and recipient bacteria at the family level (Spearman's correlation coefficient, CC)

Rank	Lipids vs Bacteria	CC	P value
**1**	PS_Staphylococcaceae	0.64	0.001
**2**	LysoPG_Pseudomonadaceae	0.62	0.001
**3**	PE_Corynebacteriaceae	0.61	0.001
**4**	PE_Staphylococcaceae	0.6	0.002
**5**	PE_Sphingomonadaceae	0.59	0.003
**6**	MGDG_Lactobacillaceae	0.58	0.002
**7**	DGDG_Corynebacteriaceae	-0.57	0.004
**8**	PI_Lactobacillaceae	-0.56	0.004
**9**	DGDG_Lachnospirascease	0.56	0.004
**10**	DGDG_Staphylococcaceae	-0.54	0.006
**11**	PG_Lactobacillaceae	-0.53	0.007
**12**	DGDG_Clostridiaceae	0.53	0.008
**13**	LysoPE_Corynebacteriaceae	0.52	0.008
**14**	LysoPE_Staphylococcaceae	0.52	0.009
15	PE_Bacteria.others	0.51	0.01
16	PE_Comamonadaceae	0.51	0.01
17	LysoPE_Sphingomonadaceae	0.51	0.01
18	LysoPC_Lactobacillaceae	-0.51	0.011
19	PC_Lactobacillaceae	-0.5	0.012
20	PA_Sphingomonadaceae	-0.5	0.013
21	PG_Clostridiales others	-0.49	0.014
22	DGDG_Sphingomonadaceae	-0.49	0.014
23	LysoPG_Bacteria.others	-0.48	0.017
24	PA_Corynebacteriaceae	-0.46	0.025
25	DGDG_Comamonadaceae	-0.46	0.025
26	PS_Sphingomonadaceae	0.45	0.026
27	PS_Propionibacteriaceae	0.45	0.027
28	PE_Oxalobacteraceae	0.45	0.029
29	LysoPE_Pseudomonadaceae	-0.44	0.03
30	PA_Nocardiaceae	-0.44	0.03
31	LysoPE_Rikenellaceae	0.44	0.031
32	PC_Sphingomonadaceae	0.44	0.032
33	LysoPG_Sphingomonadaceae	-0.44	0.033
34	PA_Staphylococcaceae	-0.43	0.034
35	LysoPE_Comamonadaceae	0.42	0.039
36	PE_Rikenellaceae	0.42	0.039
37	LysoPG_Rikenellaceae	-0.42	0.04
38	DGDG_Propionibacteriaceae	-0.33	0.121
39	PS_Bacteria.others	0.42	0.044
40	PC_Corynebacteriaceae	0.41	0.044
41	LysoPG_Staphylococcaceae	-0.41	0.044
42	PA_Rikenellaceae	-0.41	0.045
43	DGDG_Nocardiaceae	-0.41	0.046
44	PG_Ruminococcaceae	-0.41	0.046
45	LysoPE_Bacteria.others	0.41	0.046
46	LysoPC_Corynebacteriaceae	0.41	0.047
46	LysoPG_Corynebacteriaceae	-0.41	0.047
48	PS_Oxalobacteraceae	0.41	0.048
49	DGDG_Oxalobacteraceae	-0.4	0.05

Rank	Amino acids vs Bacteria	CC	P value
**1**	Tryptophan_Sphingomonadaceae	0.55	0.005
**2**	Tryptophan_Corynebacteriaceae	0.53	0.008
**3**	Glycine_Lactobacillaceae	0.52	0.01
4	Aspartic acid_Staphylococcaceae	0.5	0.014
5	Valine_Ruminococcaceae	-0.48	0.017
6	Cysteine_Sphingomonadaceae	0.47	0.021
7	Valine_Xanthomonadaceae	0.47	0.021
8	Valine_Comamonadaceae	0.46	0.022
9	Arginine_Sphingomonadaceae	0.46	0.024
10	Valine_Lachnospirascease	-0.46	0.024
11	Proline_Sphingomonadaceae	0.46	0.025
12	Isoleucine_Sphingomonadaceae	-0.45	0.027
13	Aspartic acid_Comamonadaceae	0.45	0.028
14	Proline_Corynebacteriaceae	0.45	0.029
15	Arginine_Corynebacteriaceae	0.44	0.03
16	Isoleucine_Corynebacteriaceae	0.44	0.03
17	Tryptophan_Staphylococcaceae	0.44	0.033
18	Tryptophan_Nocardiaceae	0.43	0.036
19	Tryptophan_Oxalobacteraceae	0.42	0.042
20	Aspartic acid_Corynebacteriaceae	0.42	0.043
21	Lysine_Sphingomonadaceae	0.42	0.044
22	Leucine_Corynebacteriaceae	0.41	0.047
23	Lysine_Corynebacteriaceae	0.41	0.048

Supplementary Table 9 Primer sequences used for PCR

Primers	Forward (5'-3')	Reverse (5'-3')
hPD-L1	AGCTTCACCTGCTTCGTGAGCAT	AGGGTCATGCTGGGCTTCGAGTA
mPD-L1	GACCAGCTTTTGAAGGGAAATG	CTGGTTGATTTTGCGGTATGG
hGAPDH	GTATGACAACAGCCTCAAGAT	GTCCTTCCACGATACCAAAG
mGAPDH	GGTCGGTGTGAACGGATTTG	GGAGTCATACTGGAACATGTAG
PLC-KO-Primer A/B	TG ATGGAACAGG AACTCATGCT	TCCAACTGAT GGATCATTAC CC
PLC-KO-Primer C/D	TAGAGAT TTAGGGATTA TTACTTTAAG TA	GGT GCAGAAACTC TTCTTAAACC
PLC-KO-Fusion 1		TCCAACTGATGGATCATTACCC-CACTGATTAAGCATTGGTAACTG
PLC-KO-Fusion 2	GTAGCGGCGCATTAAGCGCGG-TAGAGAT TTAGGGATTA TTACTTTAAG TA	
AmpR	CAGTTACCAATGCTTAATCAGTG	CCGCGCTTAATGCGCCGCTAC
PLC-CHIP	CTACGCTTGGGATGGAAAGAT	GTGCATAGCCTCTCCAAGATAG
PLC	CTACGCTTGGGATGGAAAGAT	GTGCATAGCCTCTCCAAGATAG
16S Universal	CTCCTACGGGAGGCAGCAG	GTATTACCGCGGCTGCTG

Supplementary Table 10 Sequence of synthetic oligonucleotides in PD-L1 promoter

Name	Sequences (5’-3’)	Distance from TSS
PD-L1p1	GTGCCCGCGG AGGCCTGGGG TGTTCTTCCG GTGCAGGCGC<br>CTGGAGCCGG GAGGTGGGCG	-1 … -60
PD-L1p2	CAACCAATCA GCGTGGCCTT TGGCTCCCTT AAGCGCTCGG<br>GTGCCCGCGG AGGCCTGGGG	-41 … -100
PD-L1p3	GCCCCAGGAT TGGTTGGCTA TGACAGTGGA TCACGAGCTT<br>CAACCAATCA GCGTGGCCTT	-81 … -140
PD-L1p4	CAGAGCGATC AGCGCAGGGA CAGTACCTTG CTTCGGCAGA<br>GCCCCAGGAT TGGTTGGCTA	-121 … -180
PD-L1p5	CGGGGACGGT GACCGCAGCA GAGGCCTCTA GCCAGAGCTC<br>CAGAGCGATC AGCGCAGGGA	-161 … -220
PD-L1p6	CCCAGGTGTC TGTGACCCGA AACACTCACG CGACACGGTG<br>CGGGGACGGT GACCGCAGCA	-201 … -260
PD-L1p7	GCAGCTGTGC GGGACCGTGC GACGGAGCCG ACCCCTCCCT<br>CCCAGGTGTC TGTGACCCGA	-241 … -300
PD-L1p8	AAGAACCCAG AGCCCTGGCC TTCCCTGCCC AGCCAGAGCT<br>GCAGCTGTGC GGGACCGTGC	-281 … -340
PD-L1p9	AACTGGGTTG GCAATACCTG TCAGCCTCGG AGGCATCAGC<br>AAGAACCCAG AGCCCTGGCC	-321 … -380
PD-L1p10	CCAACTTGGT GACAATGGTA CAGAGAGATC ACACTCACAG<br>AACTGGGTTG GCAATACCTG	-361 … -420
PD-L1p11	GAATTCCATT TTCCTGTCCA TTGCACTCTG CAGTCTTGTC<br>CCAACTTGGT GACAATGGTA	-401 … -460
PD-L1p12	TGTTGAGCCA GTTTAGCGTC ATATAGGTAT TTCCCTAAAG<br>GAATTCCATT TTCCTGTCCA	-441 … -500
PD-L1p9A	AACTGGGTTG GCAATACCTG	-321 … -340
PD-L1p9B	GCAATACCTG TCAGCCTCGG	-331 … -350
PD-L1p9C	TCAGCCTCGG AGGCATCAGC	-341 … -360
PD-L1p9D	AGGCATCAGC AAGAACCCAG	-351 … -370
PD-L1p9E	AAGAACCCAG AGCCCTGGCC	-361 … -380
PD-L1p9C-Mut	TCATCCTCGG AGGCACTAGC	

* TSS: transcription start site.
