# Supplementary material for: Plant-nanoparticles enhance anti-PD-L1 efficacy by shaping human commensal microbiota metabolites
Source: Nat Commun. 2025 Feb 3;16:1295. doi: 10.1038/s41467-025-56498-2 (PMC11790884; doi:10.1038/s41467-025-56498-2)
Supplement: Supplementary file 2 — Description of Additional Supplementary Files [file 41467_2025_56498_MOESM2_ESM.pdf]

Description of Additional Supplementary Files

Supplementary Data 1 List of metabolites in gut of germ free C57BL/6 mice by LC-MS/MS analysis

Supplementary Data 2 List of metabolites in gut of hFB C57BL/6 mice by LC-MS/MS analysis
