# Supplementary material for: Plant-nanoparticles enhance anti-PD-L1 efficacy by shaping human commensal microbiota metabolites
Source: Nat Commun. 2025 Feb 3;16:1295. doi: 10.1038/s41467-025-56498-2 (PMC11790884; doi:10.1038/s41467-025-56498-2)
Supplement: Supplementary file 5 — Reporting Summary [file 41467_2025_56498_MOESM5_ESM.pdf]

nature portfolio

Corresponding author(s): Huang-ge Zhang

Last updated by author(s): Oct 21, 2024

Reporting Summary

Nature Portfolio wishes to improve the reproducibility of the work that we publish. This form provides structure for consistency and transparency in reporting. For further information on Nature Portfolio policies, see our Editorial Policies and the Editorial Policy Checklist.

Statistics

For all statistical analyses, confirm that the following items are present in the figure legend, table legend, main text, or Methods section.

n/a	Confirmed	
☐	☒	The exact sample size (*n*) for each experimental group/condition, given as a discrete number and unit of measurement
☐	☒	A statement on whether measurements were taken from distinct samples or whether the same sample was measured repeatedly
☐	☒	The statistical test(s) used AND whether they are one- or two-sided<br>*Only common tests should be described solely by name; describe more complex techniques in the Methods section.*
☐	☒	A description of all covariates tested
☐	☒	A description of any assumptions or corrections, such as tests of normality and adjustment for multiple comparisons
☐	☒	A full description of the statistical parameters including central tendency (e.g. means) or other basic estimates (e.g. regression coefficient) AND variation (e.g. standard deviation) or associated estimates of uncertainty (e.g. confidence intervals)
☐	☒	For null hypothesis testing, the test statistic (e.g. *F*, *t*, *r*) with confidence intervals, effect sizes, degrees of freedom and *P* value noted<br>*Give P values as exact values whenever suitable.*
☒	☐	For Bayesian analysis, information on the choice of priors and Markov chain Monte Carlo settings
☐	☒	For hierarchical and complex designs, identification of the appropriate level for tests and full reporting of outcomes
☐	☒	Estimates of effect sizes (e.g. Cohen's *d*, Pearson's *r*), indicating how they were calculated

Our web collection on statistics for biologists contains articles on many of the points above.

Software and code

Policy information about availability of computer code

Data collection	None
Data analysis	None

For manuscripts utilizing custom algorithms or software that are central to the research but not yet described in published literature, software must be made available to editors and reviewers. We strongly encourage code deposition in a community repository (e.g. GitHub). See the Nature Portfolio guidelines for submitting code & software for further information.

Data

Policy information about availability of data

All manuscripts must include a data availability statement. This statement should provide the following information, where applicable:

- Accession codes, unique identifiers, or web links for publicly available datasets
- A description of any restrictions on data availability
- For clinical datasets or third party data, please ensure that the statement adheres to our policy

All data generated or analyzed during this study are included in this published article and its supplementary information files or provided in source data file. The microRNA and 16s rDNA sequencing data were deposited in the National Center for Biotechnology Information (NCBI) Gene Expression Omnibus (GEO) database with the accession number GSE229897. (https://www.ncbi.nlm.nih.gov/geo/query/acc.cgi?acc= GSE229897). The data that support the findings of this study are available on request from the corresponding author [HG.Z and Y.T.]. Source Data are provided with this paper.

Research involving human participants, their data, or biological material

Policy information about studies with human participants or human data. See also policy information about sex, gender (identity/presentation), and sexual orientation and race, ethnicity and racism.

Reporting on sex and gender	All human samples have no consideration of sex.
Reporting on race, ethnicity, or other socially relevant groupings	No race, ethnicity or other socially relevant groupings was used in this study. Anti–PD-L1 antibody was administered (1-5 mg per kilogram of body weight) every 14 days in 6-week cycles for up to 16 cycles or until the patient had a complete response or confirmed disease progression. Objective responses were confirmed by at least one sequential tumor assessment.
Population characteristics	All human fecal samples were from healthy volunteers. 61 melanoma patients were assigned to a PD-L1 responding group (n=35) and non-responding group (n=26). Anti–PD-L1 antibody was administered (1-5 mg per kilogram of body weight) every 14 days in 6-week cycles for up to 16 cycles or until the patient had a complete response or confirmed disease progression. Objective responses were confirmed by at least one sequential tumor assessment
Recruitment	All clinical samples including tissue and fecal samples were provided by the Department of Surgery, Huai'an First People's Hospital, Huai'an, Jiangsu, China with written informed consent from patients.
Ethics oversight	Approval for the study with human fecal and tumor samples was granted by the Institute Research Ethics Committee at the Health Department of Huai'an.

Note that full information on the approval of the study protocol must also be provided in the manuscript.

Field-specific reporting

Please select the one below that is the best fit for your research. If you are not sure, read the appropriate sections before making your selection.

[x] Life sciences [ ] Behavioural & social sciences [ ] Ecological, evolutionary & environmental sciences

For a reference copy of the document with all sections, see nature.com/documents/nr-reporting-summary-flat.pdf

Life sciences study design

All studies must disclose on these points even when the disclosure is negative.

Sample size	The sample size was determined by using one-way ANOVA comparing up to four groups, for a power of 0.7, a large effect size (0.75) and a significance level of 0.05, the minimum sample size needed in each group was 4.992 (rounded = 5).
Data exclusions	NO data were excluded from the analysis.
Replication	All experiments were repeated at least three times. Data are representative of three independent experiments without conflict.
Randomization	Both animals and human subjects were randomly assigned to a control group and different experimental condition groups matched for age and sex using simple randomization.
Blinding	Double-blinded studies were used for animal and human subject studies.

Reporting for specific materials, systems and methods

We require information from authors about some types of materials, experimental systems and methods used in many studies. Here, indicate whether each material, system or method listed is relevant to your study. If you are not sure if a list item applies to your research, read the appropriate section before selecting a response.

Materials & experimental systems

n/a	Involved in the study	
[ ]	[x]	Antibodies
[ ]	[x]	Eukaryotic cell lines
[x]	[ ]	Palaeontology and archaeology
[ ]	[x]	Animals and other organisms
[x]	[ ]	Clinical data
[x]	[ ]	Dual use research of concern
[ ]	[x]	Plants

Methods

n/a	Involved in the study	
[x]	[ ]	ChIP-seq
[ ]	[x]	Flow cytometry
[x]	[ ]	MRI-based neuroimaging

Antibodies

Antibodies used	Antibodies were purchased as follows: human PD-L1 (ab205921), mouse PD-L1 (ab213480), c-myc (ab32072), cleaved-caspase-3 (ab214430), cleaved-PARP (ab203467) and GAPDH (ab9485) antibodies from Abcam. The secondary antibodies conjugated to Alex Fluor-647 were purchased from Invitrogen (Eugene, OR).
Validation	All antibodies were supplied through commercial sources and all antibodies have been tested and validated by the respective company. Antibodies used for western blot, immunocytochemistry and immunohistochemistry were validated by the molecular weight, isotype non-specific lg and subcellular localizations of target proteins.

Eukaryotic cell lines

Policy information about cell lines and Sex and Gender in Research

Cell line source(s)	C57BL/6 murine melanoma B16F10 cells were purchased from American Type Culture Collection (ATCC).
Authentication	The cell line is authenticated by provider ATCC.
Mycoplasma contamination	The cell line was tested negative for mycoplasma contamination.
Commonly misidentified lines (See ICLAC register)	None

Animals and other research organisms

Policy information about studies involving animals; ARRIVE guidelines recommended for reporting animal research, and Sex and Gender in Research

Laboratory animals	Specific-pathogen-free (SPF) C57BL/6 mice were purchased from the Jackson Laboratory (Bar Harbor, ME) and germ-free (GF) inbred C57BL/6 mice were purchased from the National Gnotobiotic Rodent Resource Center (University of North Carolina, NC, Chapel Hill, NC)
Wild animals	None
Reporting on sex	We used male mice to exclude the interference of estrogens in this study.
Field-collected samples	The mice were maintained in flexible film isolators (Taconic Farm) at the Clean Mouse Facility of the University of Louisville. Animal care was performed following the Institute for Laboratory Animal Research (ILAR) guidelines,
Ethics oversight	All animal experiments were conducted in accordance with protocols approved by the University of Louisville Institutional Animal Care and Use Committee (Louisville, KY).

Note that full information on the approval of the study protocol must also be provided in the manuscript.

Plants

Seed stocks	No seed stocks were used in this study.
Novel plant genotypes	None
Authentication	All plants used in this study are dietary plants and were purchased from a local supermarket.

Flow Cytometry

Plots

Confirm that:

☒ The axis labels state the marker and fluorochrome used (e.g. CD4-FITC).

☒ The axis scales are clearly visible. Include numbers along axes only for bottom left plot of group (a 'group' is an analysis of identical markers).

☒ All plots are contour plots with outliers or pseudocolor plots.

☒ A numerical value for number of cells or percentage (with statistics) is provided.

Methodology

Sample preparation	Isolated cells were incubated with blocking solution (0.5% BSA/PBS) for 15 min at 4°C. For surface staining, cells were stained with various antibodies at room temperature for 1 h and then washed with PBS. Washed cells were stained for 40 min at 4°C with the appropriate fluorochrome-conjugated antibodies in PBS with 2% FBS.
Instrument	Data were acquired using a BD FACSCalibur flow cytometer (BD Biosciences, San Jose, CA)
Software	The data were collected using BD FACStation software and analyzed using FlowJo software (Tree Star Inc., Ashland, OR).
Cell population abundance	0.5-1.0 x 10^6 cells/ml were used to analyze the abundance of cell populations. 10^5 cells were collected for analysis of cell frequency.
Gating strategy	Forward scatter (FSC) and side scatter (SSC) are used to distinguish cell populations based on their size and granularity respectively. CD8 positive cells were selected with the histogram plot for further PD-1 and cytokine analysis.

☒ Tick this box to confirm that a figure exemplifying the gating strategy is provided in the Supplementary Information.
